# YY1 suppresses FEN1 over-expression and drug resistance in breast cancer

**DOI:** 10.1186/s12885-015-1043-1

**Published:** 2015-02-13

**Authors:** Jianwei Wang, Lina Zhou, Zhi Li, Ting Zhang, Wenpeng Liu, Zheng Liu, Yate-Ching Yuan, Fan Su, Lu Xu, Yan Wang, Xiaotong Zhou, Hong Xu, Yuejin Hua, Ying-Jie Wang, Li Zheng, Yue-E Teng, Binghui Shen

**Affiliations:** 1 College of Life Sciences, Zhejiang University, Hangzhou, China; 2 Departments of Radiation Biology and Molecular Medicine, Beckman Research Institute of City of Hope, 1500 East Duarte Road, Duarte, California 91010 USA; 3 Departments of Medical Oncology and Thoracic Surgery, The First Hospital of China Medical University, No. 155 North Nanjing Street, Heping District, Shenyang, 110001 China; 4 College of Agricultural Sciences and Biotechnology, Zhejiang University, Hangzhou, China; 5 School of Medicine, Zhejiang University, Hangzhou, China

**Keywords:** Flap endonuclease 1 (FEN1), YY1, Over-expression, Promoter, Drug resistance

## Abstract

**Background:**

Drug resistance is a major challenge in cancer therapeutics. Abundant evidence indicates that DNA repair systems are enhanced after repetitive chemotherapeutic treatments, rendering cancers cells drug-resistant. Flap endonuclease 1 (FEN1) plays critical roles in DNA replication and repair and in counteracting replication stress, which is a key mechanism for many chemotherapeutic drugs to kill cancer cells. FEN1 was previously shown to be upregulated in response to DNA damaging agents. However, it is unclear about the transcription factors that regulate FEN1 expression in human cancer. More importantly, it is unknown whether up-regulation of FEN1 has an adverse impact on the prognosis of chemotherapeutic treatments of human cancers.

**Methods:**

To reveal regulation mechanism of FEN1 expression, we search and identify FEN1 transcription factors or repressors and investigate their function on FEN1 expression by using a combination of biochemical, molecular, and cellular approaches. Furthermore, to gain insights into the impact of FEN1 levels on the response of human cancer to therapeutic treatments, we determine FEN1 levels in human breast cancer specimens and correlate them to the response to treatments and the survivorship of corresponding breast cancer patients.

**Results:**

We observe that FEN1 is significantly up-regulated upon treatment of chemotherapeutic drugs such as mitomycin C (MMC) and Taxol in breast cancer cells. We identify that the transcription factor/repressor YY1 binds to the *FEN1* promoter and suppresses the expression of *FEN1* gene. In response to the drug treatments, YY1 is dissociated from the FEN1 promoter region leading over-expression of *FEN1*. Overexpression of YY1 in the cells results in down-regulation of FEN1 and sensitization of the cancer cells to MMC or taxol. Furthermore, we observe that the level of *FEN1* is inversely correlated with cancer drug and radiation resistance and with survivorship in breast cancer patients.

**Conclusion:**

Altogether, our current data indicate that YY1 is a transcription repressor of FEN1 regulating FEN1 levels in response to DNA damaging agents. FEN1 is up-regulated in human breast cancer and its levels inversely correlated with cancer drug and radiation resistance and with survivorship in breast cancer patients.

**Electronic supplementary material:**

The online version of this article (doi:10.1186/s12885-015-1043-1) contains supplementary material, which is available to authorized users.

## Background

Chemotherapy is a major therapeutic treatment for cancer. The effectiveness of most current chemotherapeutic drugs for cancer depends on the ability to induce DNA damage in hyper-proliferating cancer cells, which have inadequate DNA repair capacity. However, the development of multidrug resistance (MDR) in cancer cells poses a major challenge to chemotherapy and greatly limits the anti-cancer efficacy of chemotherapeutic drugs [1,2]. Such resistance arises in cancer cells and cancer stem-like-cells not only because of the alteration in drug transport and metabolism that results in low level of anticancer efficacy, but also because of the increased tolerance for DNA lesion and enhanced DNA replication and repair capacity [1-5]. DNA repair pathways, including base excision repair (BER), nucleotide excision repair (NER), mismatch repair (MMR), interstrand crosslink repair (ICL), non-homologous end joining (NHEJ), and homologous recombination (HR), have been implicated to play important roles in modulating the response of human cancer to chemotherapy. Previous studies have shown that cancer cells resistant to chemotherapeutic drugs have abnormally high DNA repair capacity [6]. Furthermore, inhibition of DNA repair has successfully sensitized the cancer cells to cytotoxic killing by chemotherapeutic drugs [7].

Efficient DNA damage repair partly depends on the structure-specific nuclease family members, which remove damaged bases or nucleotides and process various DNA intermediate structures. Flag endonuclease 1 (FEN1) is an important member of this family, playing a pivotal role in DNA replication and repair [8-10]. Although FEN1 was once widely considered a tumor suppresser [11] based on its role in the maintenance of genomic stability through Okazaki fragment maturation, long-patch base excision repair [12-14], rescue of the stalled replication fork [15], and telomere maintenance [16-19], accumulated evidences now indicate that FEN1 is required for tumor progression [20-23]. Its expression is up-regulated in response to treatments with anti-cancer drugs or with radiation admission, thus enhancing DNA repair pathways and contributing to cancer cells’ survival under genome toxic stresses [7,22,24]. Using cancer profiling array and immune-histochemistry, we have previously found that FEN1 is clearly over-expressed in breast cancer tissues [22]. In addition, FEN1 is also highly expressed in lung [25] and gastric cancer cell lines [26], as well as prostates cancer [21,27], neuroblastomas [28], testis, lung, and brain tumors *in situ* [7]. Interestingly, FEN1 is significantly up-regulated in mouse fibroblasts in a p53-dependent manner under genome toxic stresses such as exposure to UV-C [29] and DNA-alkylating drugs [30]. Recently, Nikolova et al. showed that down-regulation of *FEN1* expression by siRNA in LN308 glioma cells increased the cells’ damage-sensitivity to methylating agents such as methyl methane-sulfonate and temozolomide [7]. All evidences suggest that alteration of FEN1 expression-level corresponds to cellular responses to chemotherapy or radiation. However, the underlying mechanisms that up-regulates FEN1 upon drug treatment and confers the drug resistance to cancer cells remain unclear.

Here, we identify multiple potential transcription factor binding sites in the *FEN1* promoter region. Using DNA fragments corresponding to *FEN1* promoter regions, we pulled down the proteins bounded to the DNA fragments in the cell crude extracts prepared from cells grown under normal cell culture conditions and identified them using mass spectrometry. One of the outstanding transcription factors that we have identified is Ying Yang 1(YY1), which plays an important role in divergent biologic processes such as embryogenesis, differentiation, cellular proliferation and cancer progression [31,32]. YY1 is well known for its dual roles in regulating gene expression, either as activator or repressor, depending upon the context in which it binds to [33-36]. In this study, we found that YY1 is a repressor for FEN1 expression. In response to DNA damaging agents, YY1 dissociated from *FEN1* promoter, leading to up-regulation of FEN1 for DNA repair. Furthermore, we revealed that the elevated FEN1 level promotes the efficiency of DNA repair, which consequently leads to drug resistance and poor prognostics.

## Methods

### Design of the biotinylated DNA probes

**Figure 1:**
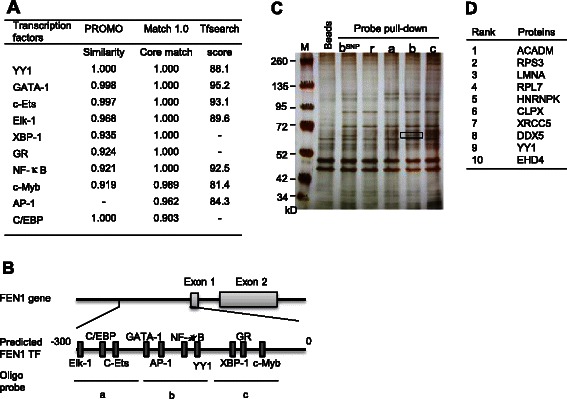
**Identification of YY1 as a potential transcription regulator for FEN1. A**. Top 10 hits of the transcription factors (TFs) that were predicted by TF Research Web sites: Match1.0-public, PROMO, and TFSEARCH. **B**. The oligo probes were designed to cover different regions of the predicted *FEN1* promoter. Probes a, b, and c correspond to the region −290 to −230, −150 to −90, and −60 to 0, respectively. **C**. The silver staining image of oligo-pulled-down assays using HeLa cell extracts. b_SNP_: probe b with three SNP sites, r: a probe with random DNA sequences. The unique band, which is indicated by a box, was subjected to MS analysis. **D**. Top 10 hits of the MS analysis of the unique protein band as specified in Panel **C**.

We predicted the potential transcriptional factors bound to the −300/+70 fragment of hFEN1’s promoter with the following databases: Match1.0-public, TESS, and TFSEARCH. We found 200 transcriptional factors including NF-kB, YY1, p300, USF1, NRF-2 (Figure 1A). We designed the probes covering the majority of the transcription factor binding sites. The sequences of all of the probes including Probe a, Probe b, Probe c, Probe bSNP and Probe R, which are random sequence controls, are listed in Additional file 1: Table S6. These probes were synthesized by Sangon Biotech (Shanghai, China).

### Preparation of nuclear extracts

Crude nuclear extracts from HeLa cell were prepared according to a procedure previously described [37]. In brief, the harvested cells were washed twice with ice cold PBS and resuspended in 5 package cell-volume of buffer A (10 mM HEPES [pH 7.9], 1.5 mM MgCl_2_, 10 mM KCl, 0.5 mM dithiothreitol) containing protease inhibitor cocktail (Roche, Indianapolis, IN, USA). NP-40 was added to a final concentration of 0.5% and kept on ice for 10 min. The nuclear pellet was obtained by centrifugation at 1500 rpm for 4 min at 4°C. Then the pellet was washed by 5 package cell-volume buffer A without NP-40. Supernatant was removed and the pellet was resuspended in 1 package cell volume of buffer (20 mM HEPES, pH 7.9, 1.5 mM MgCl_2_, 420 mM NaCl, 0.2 mM EDTA, 2.5% glycerol) with protease inhibitors. The mixture was sonicated for 5 s and kept on ice for 60 min. and vertex briefly every 10 mins. The nuclear extracts (supernatants) were obtained with centrifugation for 10 min at 12,000 g and 4°C.

### Biotinylated DNA probe pull down assay and mass spectrometry

Biotinylated DNA pull-down assay was performed as previously described [37,38] with modifications. 100 μl (50 nM) of biotinylated probe were incubated with 200 μl HeLa nuclear extracts in 700 μl binding buffer (25 mM Tris, 150 mM NaCl, pH 7.2) with protease inhibitor cocktails and phosphatase inhibitor cocktails (Roche, Indianapolis, IN, USA) for 30 minutes at room temperature with gentle rotation. 20 μl streptavidin conjugated agarose (Pierce, Rockford, IL, USA) was washed with PBS (pH 7.4) and was added into the DNA-protein complexes for 1 hour at room temperature with gentle rotation. Agarose bead-DNA-protein complexes were washed three times with ice cold binding buffers and then were eluted in SDS-PAGE loading buffer by heating at 95°C. All samples were loaded onto 12% SDS-PAGE, followed by silver staining with silver stain kit (Beyotime, China). The unique protein band as shown in Figure 1C was excised and subjected to mass spectrometry analysis (Protein Mass Spectrometry Analysis Center, Institutes of Biochemistry and Cell Biology, Chinese Academy of Sciences, Shanghai).

### Protein expression and purification

3xFlag tagged YY1 was expressed in 293 T cells and purified following a published protocol [39]. Briefly, pCMV7.0-YY1, which encodes the recombinant 3xFlag tagged YY1, was transfected into 293 T cells. After removal of the transfection reagent, the cells were incubated in fresh DMEM medium for 48 h and then harvested. The cells were lysed in 1 ml lysis buffer (Tris 50 mM, 500 mM NaCl, 10% Glycerol, 0.5% NP40, 1 mM DTT, 1 mM EDTA, 1 mM PMSF and protease inhibitor cocktail), and the lysates were centrifuged at 20,000 g for 10 min. The supernatant was incubated with equilibrated 25 μl Anti-Flag M2 magnetic beads (Sigma, St Louis, MO, USA) for 12 h. After it was extensively washed with lysis buffer, the 3xFlag tagged YY1 was eluted with 50 μl 2 mg/ml 3xFlag peptide (Genescript, China). The primers used to amplify the YY1 cDNA are listed in Additional file 1: Table S6.

### Electrophoretic mobility shift assay (EMSA)

EMSA was performed as described previously [40] by using the Light Shift chemiluminescent EMSA kit (Thermo Fisher Scientific, Wilmington, DE, USA), purified recombinant YY1 protein and the biotin-labeled double strand DNA. These probes, which represent the *FEN1* promoter regions, include negative control Probe N, positive control Probe P, WT FEN1 and MUT *FEN1*. The positive control probe (Probe P) is the same Probe as the Probe b used in the biotinylated DNA pull-down assay. The MUT FEN1 probe contains two mutated nucleotide residues indicated with low case. These probes are listed in Additional file 1: Table S6.

### Chromatin immunoprecipitation (ChIP)

ChIP assay was performed as described previously [40]. The rabbit anti-YY1 antibody was purchased from Santa Cruz Biotechnology (Santa Cruz Biotechnology, Dallas, TX, USA). The protein A/G agarose beads were purchased from Pierce (Pierce, Rockford, IL, USA) and mouse IgG conjugated with magnetic beads were purchased from Cell Signaling Technology (Cell Signaling Technology, Danvers, MA, USA) as the negative control. Besides the control IgG, the amount of ACTB and FEN1 CDS DNA fragment that was precipitated and analyzed under same conditions served as an additional control for specificity of the binding between the ChIP antibodies and their target genes. ChIP primers for the FEN1 promoter, FEN1CDS and ACTB, as a control, are listed in Additional file 1: Table S6.

### Cell culture, transfection, treatment, and flow cytometry

The 293 T, HeLa, MCF-7, MDA-MB-231 cells were obtained from ATCC. Cells were cultured in DMEM (Hyclone, Logan, UT, USA) supplemented with 10% fetal bovine serum (Pufei, China). 1 × 10^6^ MDA-MB-231 or MCF7 cells were seeded in 6 well-plate for 24 h at 37°C, 5% CO_2_, then treated with 5 μM Mytomycine C (MMC) (Sigma, St Louis, MO, USA) for 1 h. After treatment, cells were collected 9 and 16 hours later for RT-PCR and Western blotting to detect the YY1 and FEN1 protein and mRNA levels, respectively. In parallel, cells were treated with Taxol (Melone, China) in a concentration of 20 nM for 24 h and were then collected for RT-PCR and Western blotting.

The transfections were carried out according to standard procedures using SuperFectin II DNA Transfection Reagent (Pufei, China) and the EGFP intensity was measured with the Cytomics TM FC 500 Flow Cytometer System (Beckman Coulter, Pasadena, CA). To detect the effects of the YY1 level in cellular response to the drugs, 239 T cells were transfected with pcDNA3.1-YY1. The cell survival fractions at different time points were measured by cell counting.

### Western blotting

Western blotting analysis was performed according to standard procedures using ECL detection substrate (Pierce, Rockford, IL, USA) and the blot was exposed to the Tannon 5200 System for visualization. The antibodies used in our studies were the rabbit polyclonal anti-YY1 antibody (Santa Cruz), the rabbit monoclonal anti-FEN1 antibody (Novus Biologicals, Littleton, CO, USA), the Horseradish peroxidase (HRP)-conjugated anti-GAPDH (GenScript, China), and the Horseradish peroxidase (HRP)-conjugated anti-rabbit secondary antibody (Pierce, Rockford, IL, USA).

### RT-PCR analysis

Total mRNA was isolated using TRIzol reagent (Life Technologies, Carlsbad, CA, USA). Reverse transcription reaction was performed using PrimeScript RT reagent kit (TaKaRa, Japan) according to the manufacturer’s instructions. qRT-PCR was performed in a MJ Chromo 4 (Bio-Rad) by using a reaction mixture with Platinum SYBR qPCR SuperMix-UDG (Invitrogen, Carlsbad, CA, USA). All the PCR amplification was performed in triplicate and repeated in three independent experiments. The sequence for all of the primers for human *FEN1*, human *YY1*, and the internal control of human GAPDH and EGFP are listed in Additional file 1: Table S6.

### Disease free survival analyses based on the data available in the literature

FEN1 survival analyses were determined based on Ivshina et al. [41]. In their study, the gene expression was profiled with 347 primary invasive breast tumors using Affymetrix microarray. Data were deposited to Gene Expression Omnibus (GEO) database (GSE4922). The FEN1 expression ‘high’ and ‘low’ groups were segregated based on median expression values. Kaplan-Meier survival analysis was used to determine the survival differences between ‘high’ and ‘low’ expression, visualized by Kaplan-Meier plots and compared using Cox regression analysis, with p-values calculated by log-rank test using the Survival package in R [42]. Survival analyses were performed on all patients, including ER+ subgroups, ER- subgroups and ER negative and lymph node negative (ER-LN-) groups respectively for clinical interest.

### Patient information and tumor specimens for prognostic outcome analysis

The use of specimens from human subjects was approved by the Ethics Committee of China Medical University (CMU). A total of 288 primary breast cancer patients from the archives of the Department of Pathology in the First Hospital of CMU were initially recruited in the current retrospective study. All patients included in the study were the ones who had surgery between May 1995 and December 2009. Patients were selected into the study based on the availability of complete clinical medical records, follow-up data and an adequate number of paraffin-embedded tissue blocks.

The current study includes follow-up data available as of Oct. 2013. The medium follow-up duration was 90.8 months with a range from 11.7 to 167.4 months. The overall survival (OS) was set on the period from the date of surgery to death or to the most recent clinic visit. The disease-free survival (DFS) was set on the period from the date of surgery to recurrence, death, or to the most recent clinic visit. The complete demographic and clinical data were collected retrospectively. Formalin-fixed, paraffin-embedded tumor specimens were obtained from the archives of the Department of Pathology of the First Hospital of CMU and three pathologists examined all the specimens to confirm histopathological features. The tumors were staged according to the criteria set by the American Joint Committee on Cancer (AJCC) stage (The 7th edition).

### Tissue microarray and IHC

A tissue microarray was constructed in collaboration with Shanghai Biochip (Shanghai, China). Two punch cores of 1.0 mm were taken from each patient sample from the non-necrotic area of tumor foci. IHC protocols are described in detail [22]. After they were counterstained with Meyer’s haematoxylin, the sections were observed under a light microscope by an experienced pathologist with cytoplasmical or nuclear patches of brown scored as FEN1-positive. For YY1, a cell was considered positive if there were brown patches in nuclei. A scale was applied to quantify the extent of expression: 0 = no detectable or only trace staining, 1 = weak expression, 2 = strong expression. Score 0 was considered as “low expression”, and score 1 and 2 were considered as “high expression”.

### Prognostic outcome analysis

A Spearman’s correlation test was used to assess relationships between variables. Survival curves were plotted by the Kaplan-Meier (KM) method and compared with the log-rank test. All the clinicopathological variables listed in Additional file 1: Table S1 were included in a multivariate Cox model that was modified in a backward stepwise manner to select the variables that carried prognostic value independent of each other. The associations with FEN1, YY1 or combination of the two and clinical outcomes were assessed using an unadjusted model and after adjusting for the selected variables in the previous step. Hazard ratios (HR) and 95% confidence intervals (CI) were estimated. The cutoff values were selected on quartiles, and the frequency of distribution of variables, the size, and the number of events in each subgroup were also considered. Groups with similar survival were merged. All statistical tests were two-tailed with a P < 0.05 considered significant. SPSS statistical software (SPSS, Inc.) was used for the above statistical analysis.

## Results

### Identification and validation of transcription factor YY1 binding to FEN1 promoter

We previously showed that the −458 to +278 region of the FEN1 gene promoter is essential to drive its expression [22]. To investigate which transcription factors regulate *FEN1* expression, we first employed bioinformatics studies using the Match 1.0-public, TESS, and TESEARCH databases to predict the potential transcriptional factor binding sites in the region from −300 to +70 nt of h*FEN1*’s promoter. These analyses revealed the consensus binding elements for nearly 200 transcription factors including NF-kB and YY-1 (Figure 1A). To experimentally determine whether these transcription factors indeed bind to the *FEN1* promoter, we designed three probes (a, b, and c) to cover different regions of the human *FEN1* promoter (Figure 1B). The probes a, b, and c correspond to the promoter regions from −290 to −230, −150 to −90, and −60 to 0, respectively. In addition, probe b_SNP_ contains the same region of −150 to −90 as probe b, but includes three single nucleotide polymorphisms that have been reported in NCBI database. Using these probes, we pulled down the proteins bounded to the DNA fragments in the cell crude extracts prepared from HeLa cells grown under normal cell culture conditions. On the silver stained SDS-PAGE, we observed a unique band (boxed) in the lanes of the probe b pulled-down proteins (Figure 1C). The band was also present in the lane of the probe b_SNP_ pulled-down proteins, indicating SNPs do not influence the binding capacity of the contained transcriptional factors. To reveal what proteins correspond to this band, we excised the band and identified the proteins with mass spectrometry analyses. Transcriptional factor YY1 was among the top 10 hits (Figure 1D).

**Figure 2:**
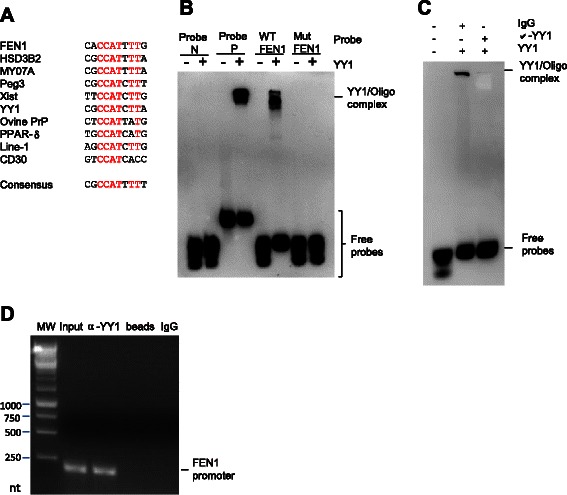
**YY1 binds to the conserved YY1 binding motif in the FEN1 promoter region. A**. Sequence alignment of the conserved YY1 binding motif in different proteins. **B**. EMSA analysis of YY1 binding to the YY1 binding motif in the *FEN1* promoter. Recombinant YY1 was incubated with different biotin-labeled DNA probes. The sequences of the Probe N, Probe P, WT FEN1and MUT FEN1 can be found in Additional file 1: Table S6. The free probe and YY1/DNA complex were resolved in 5% native PAGE. **C***.* EMSA assay on YY1 and FEN1 oligo in the presence of non-specific IgG or the anti-YY1 antibody. **D***.* ChIP analysis of YY1 binding to the FEN1 promoter region. Specific YY1-bound DNA in MCF7 cell extracts was pulled down by an anti-YY1 antibody. The YY1-bound FEN1 sequence was amplified by PCR. The sequence for the *FEN1* promoter specific primer can be found in the Additional file 1: Table S6 as FEN1 (YY1). The PCR product was analyzed by 1% agarose electrophoresis.

YY1 is a ubiquitously distributed transcriptional factor that regulates numerous gene expressions [43-47]. We found that the binding site for YY1 on *FEN1* promoter was conserved based on the sequence alignment of the predicted YY1 binding motif to the binding sites from various genes (Figure 2A). To validate whether YY1 indeed binds to the predicted YY1 binding site on the *FEN1* promoter region, we performed the electrophoretic mobility shift assay (EMSA) using the purified recombinant YY1 protein and the DNA probe, a 29 base pair oligonucleotide covering the predicted YY1 binding site. We found that YY1 effectively binds to the wild type probe, forming the YY1/DNA complex, which displayed a retarded migration compared to the free probe. Furthermore, substitution of the conserved “C” and “T” nucleotide with “G” and “A” abolished the formation of the YY1/DNA complex (Figure 2B). To further verify the binding of YY1 to the DNA sequence in the FEN1 promoter region, we added non-specific IgG or anti-YY1 anti-body to the binding reaction with YY1 and WT FEN1 sequence. Addition of anti-YY1 but not non-specific IgG diminished the YY1-oligo complex (Figure 2C), suggesting that YY1 specifically bound to the oligo sequence of FEN1 promoter. We then investigated whether YY1 bound to the *FEN1* promoter region in MCF7 breast cancer cells by conducting a chromatin immune-precipitation-PCR (ChIP-PCR) and showed that the FEN1 promoter was specifically pulled-down by an YY1-specific antibody but not the control antibody (Figure 2D). The results all suggest that transcriptional factor YY1 binds to the *FEN1* promoter.

### Anti-cancer drugs release the YY1 suppression to FEN1 leading to its over-expression and drug resistance

**Figure 3:**
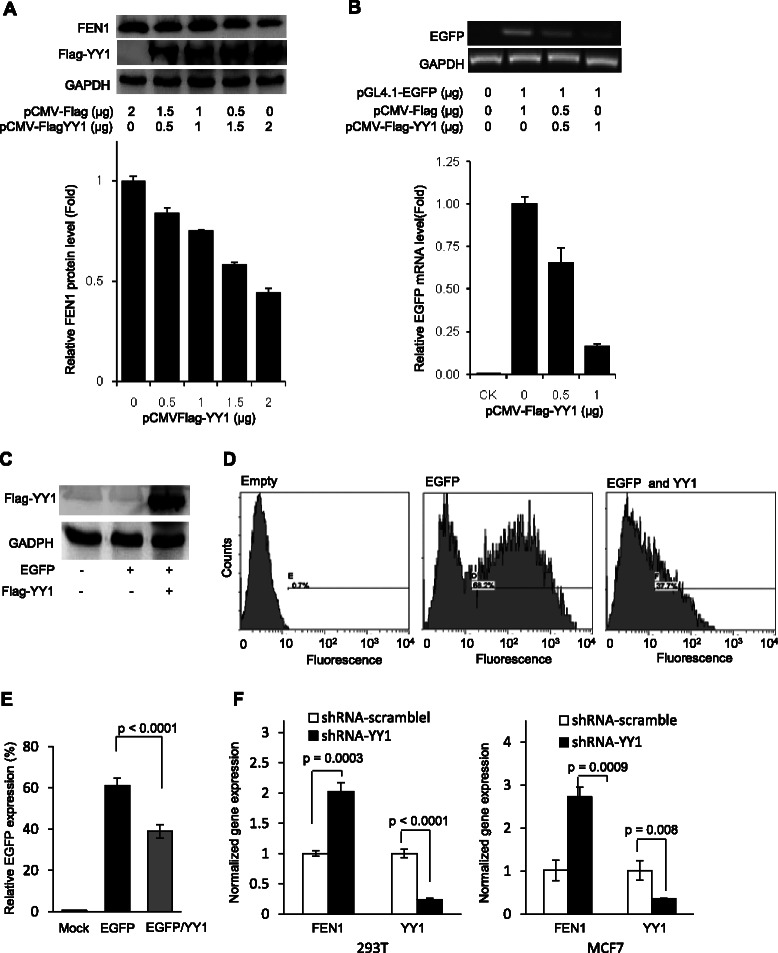
**Overexpression of YY1 inhibits FEN1 promoter-driven protein expression. A**. YY1 was overexpressed in 293 T cells and its impact on the FEN1 protein level was evaluated by western blot using the anti-Flag or anti-FEN1 antibody. **B**. The pCMV-Flag-YY1 expression vector and the pGL4.0-FEN1 promoter-EGFP vector, or pGL4.0 EGFP vector was co-transfected into 293 T cells. The EGFP expression was detected by semi-quantitative PCR (Upper panel) and quantitative PCR (lower panel). **C**. The overexpression of Flag-tagged YY1 was confirmed by western blot using the anti-Flag antibody. **D** and **E**. EGFP protein levels with or without YY1 overexpression was measured by FACS. Panel **D** shows the representative FACS images. Panel **E** is the quantification of FACS. Values are means ± s.d. of four independent experiments. p value was calculated by the two-tail student’s t-test. **F**. Knockdown of YY1 in 293 T (Left Panel) and MCF7 (right panel) cells. The YY1 and FEN1 expression was measured by quantitative PCR. The mRNA level was normalized with corresponding mRNA level of GADPH, and the normalized mRNA level of YY1 or FEN1 in the cells treated with control siRNA was arbitrarily set as 1. Values are means ± s.d. of three independent experiments. p value was calculated by the two-tail student’s t-test.

YY1 is a multifunctional protein and can work as either a gene expression repressor or an activator [35,48]. To determine the roles of YY1 in regulation of FEN1 expression, we exogenously overexpressed YY1 in 293 T cells and evaluated the FEN1 protein level. We found that the protein level of endogenous FEN1 gradually decreased as the amounts of the plasmid DNA transfected into 293 T cells increased (Figure 3A). We next examined whether YY1 bound to the *FEN1* promoter region and suppressed the gene expression. We sub-cloned the *FEN1* promoter into the pGL4.0 plasmid, so that the expression of the EGFP reporter gene was only driven by the *FEN1* promoter. The Flag-tagged YY1 expression vector and the pGL4.0-*FEN1* promoter-driven *EGFP* vector were co-transfected into 293 T cells. The overexpression of Flag-tagged YY1 was confirmed by PCR and western blot (Figure 3B and C). We then measured the *EGFP* mRNA level by qPCR and the EGFP protein by flow cytometry. Our data indicated that the ectopic over-expression of YY1 in 293 T cells considerably reduced *EGFP* mRNA and protein levels (Figure 3B, D and E). Next, we determined if a decrease in YY1 level resulted in up-regulation of FEN1 expression. We knocked down YY1 in 293 T or MCF7 cells by shRNA specific against YY1 sequences. We found that knockdown of YY1 was associated with significant increase in FEN1 expression level in both 293 T and MCF7 cells (Figure 3F). Similar phenomenon was observed in HeLa and U251 cancer cells.

**Figure 4:**
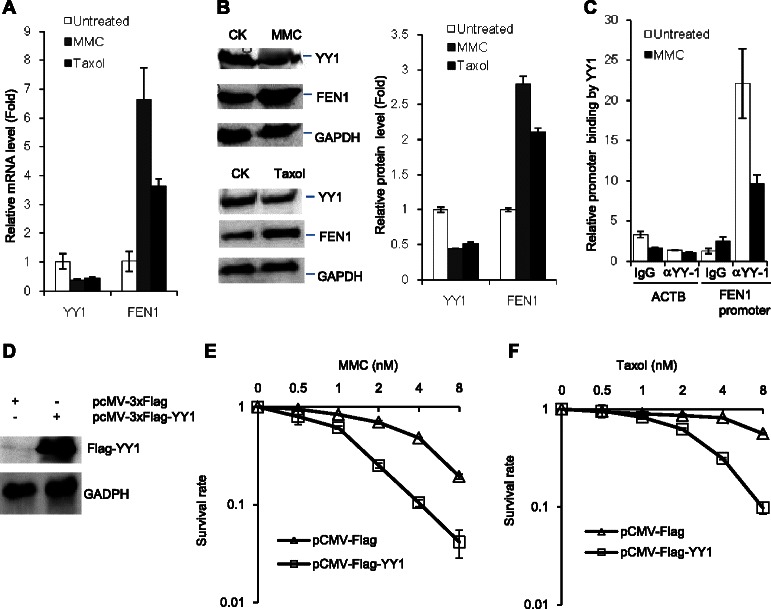
**DNA damaging agents MMC and Taxol inhibit YY1 expression but induce FEN1 expression. A**. and **B**. YY1 and FEN1 expression in MDA-MB-231 breast cancer cell line in response to the MMC and Taxol treatment. The mRNA level **(A)** and protein level **(B)** were measured by quantitative PCR and Western blot. The left panel in **B** showed the quantification of Western blot results. All experiments were independent carried out at least three times. **C**. Analysis of YY1 binding to the *FEN1* promoter in response to the MMC treatment. Cells were treated with MMC, and the level of YY1-bound FEN1 promoter was evaluated by the ChIP assay. The lowest DNA staining density is arbitrarily set as 1. **D**. western blot confirmed the overexpression of Flag-tagged YY1 in 293 T cells. The β-actin(ACTB) was used as control. **E**. and **F**. The survivorship of 293 T cell and 293 T cell harboring a YY1 expression plasmid, pCMV-Flag-YY1, or the empty vector, under treatment of mytomycine **C** (MMC) **(Panel E)** or taxol **(Panel F)**. In both panels, the cells were treated with indicated concentrations of MMC or taxol for 48 hours. The number of survival cells was counted. The survival rate of the untreated cells with or without YY1 overexpression was arbitrarily set as 1.

We then tested whether DNA damaging agents and chemotherapeutic drugs relieve such a restraint, leading to induction of FEN1 expression. We treated the breast cancer cell line MDA-MB-231 with mitomycin C (MMC) and Taxol and performed qPCR and Western blotting to analyze the gene expression of *YY1* and *FEN1*. We found that in response to treatments with MMC and Taxol, the mRNA level of *YY1* was down-regulated by more than 2 folds, while the mRNA level of *FEN1* was up-regulated by 3 to 6 folds (Figure 4A and B). We consistently observed that the YY1 protein level was reduced by approximate 2 folds, while the protein level of FEN1 increased by more than 2 folds. In addition, we tested whether the drug treatment also impairs the binding of the transcription factor to the *FEN1* promoter. Indeed, our ChIP analyses indicated that the amount of YY1 bound to the *FEN1* promoter reduced by 2 folds upon the MMC treatment (Figure 4C). Furthermore, when we overexpressed the Flag-tagged YY1 in 293 T cells (Figure 4D), we observed that the cells harboring this expression plasmid became more sensitive to both MMC and Taxol treatment (Figure 4E and F).

**Figure 5:**
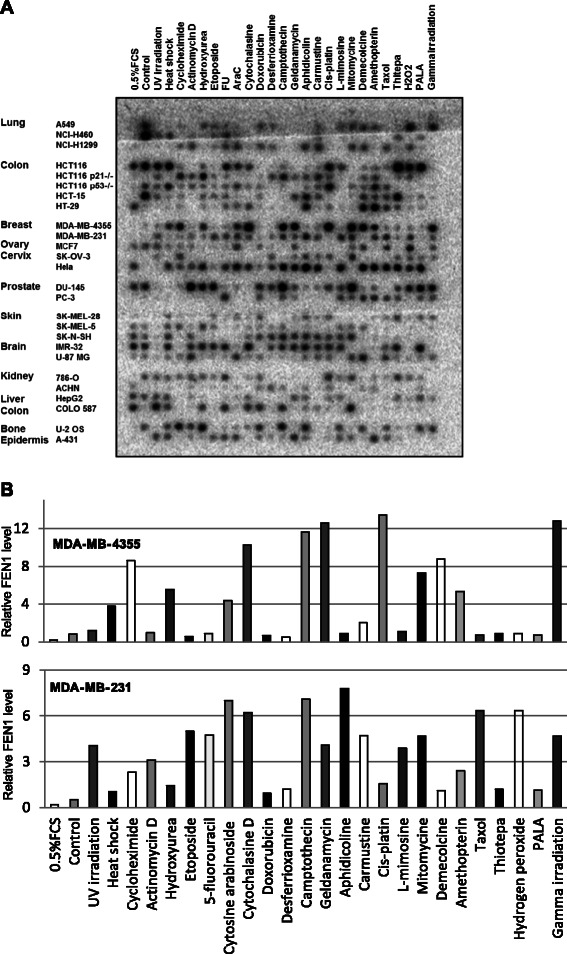
**FEN1 gene expression in response to chemotherapeutic drug and DNA damage agent treatments. A**. Macro-image of hybridization of the ^32^-P-labeled FEN1 ORF DNA fragment with the expression array, which contains cDNA from different cells lines treated with different DNA-damaging agents. In the control hybridization, ^32^-P-labeled ubiquitin ORF DNA fragment was used [38]. **B**. The relative fold changes of the density of the hybridized spots. All *FEN1* hybridization signals were normalized with corresponding ubiquitin signals. In each cell line, the normalized signal in the untreated control sample was arbitrarily set as 1, and the fold change was calculated by comparing the normalized signal in a specific sample to that of the untreated control sample.

To support the notion that different DNA damage agents and therapeutic drugs induce FEN1 gene expression, we employed an expression array of 26 cancer cell lines in 13 major categories that have been treated with 25 different DNA-damaging agents and therapeutic drugs (Figure 5A). The fact that FEN1 expression was high in breast cancer cell lines was consistent with our published data [22]. The Northern dot blotting results showed that FEN1 expression levels in breast cancer cell lines, MDA-MB-4355 and MDA-MB-231, increased significantly (by more than 8 folds) after the treatment with DNA-damaging agents, such as camptothecin, cytochalasin D, MMC, and gamma irradiation (Figure 5A and B). However, other agents such as Etoposide, 5-fluorouracil, Aphidicoline and Taxol, induced the FEN1 expression in MDA-MB-231, but not in MDA-MB-4355 (Figure 5A and B).

### Breast cancer patients with low expression of YY1 and high expression of FEN1 have poor prognostics

**Figure 6:**
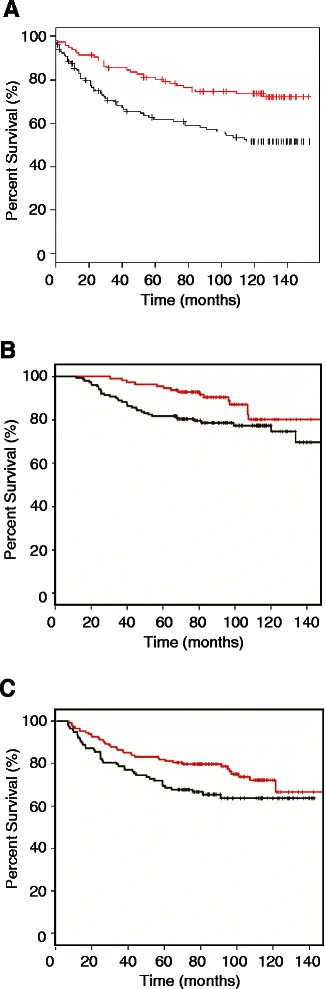
**Associations between FEN1, YY1 protein, or their combination and OS or DFS. A**. FEN1 Kaplan Meier survival plot with breast cancer patient cohort in the Ivshina data base. The black line indicates FEN1 high expression while the red line indicates FEN1 low expression (‘high’ and ‘low’ determined by median expression). Patients with FEN1 high expression: 132; patients with FEN1 low expression: 117, Log-rank p = 0.0007. **B**. Low expression of YY1 and high expression of FEN1 and OS in the CMU cohort. The black line indicates YY1 low but FEN1 high expression and red line indicates other types. Patients with YY1 low but FEN1 high expression: 154; patients with other types: 113, Log-rank p = 0.027. **C**. Low expression of YY1 and high expression of FEN1 and DFS in the CMU cohort. The black line indicates YY1 low but FEN1 high expression and red line indicates other types. Patients with YY1 low but FEN1 high expression: 154; patients with other type: 113, Log-rank p = 0.048.

Seeking the relevance between FEN1 expression and cancer patient outcomes, we performed survival analysis using 5 different breast cancer patient cohorts, namely Ivshina [41], Huang [49], Pawitan [50], Sotiriou [51], and Wang [52], all of which are available in the literature. For the data from the Ivshina [41], patients were grouped into High-Risk and Low-Risk subgroups based on 2-mean categorical clustering of selected significant genes for Kaplan-Meier survival analysis [41] with high and low expression levels of *FEN1* gene to measure the number of patients living for a certain amount of time after the treatment. Kaplan-Meier analyses revealed that the under-expression of *FEN1* measured by the mRNA level was correlated with better disease free survival (DFS) outcome. For overall 249 breast tumor samples (p = 0.0007), 211 of ER+ subgroups (p = 0.005), 34 of ER- subgroups (p = 0.03), 20 of ER-LN- subgroups (p = 0.007) all showed an inverse correlation of *FEN1* gene expression with DFS (Figure 6A). The inverse correlation was also validated with other 4 large breast cancer cohorts (Huang et al. (n = 89; p = 0.004) [49], Pawitan et al. (n = 159; p = 5.21e-5) [50], Sotiriou et al. (n = 117 p = 0.04) [51] and Wang et al. (n = 286 p = 0.02) [52]. Interestingly, our additional patient cohort data mining indicated that the difference of the survivorship between the patients with the low expression and high expression of *FEN1* in ER- and ER-/lymph node negative patient cohorts is much larger than that in ER+ patients (Data not shown).

Further seeking the association between FEN1 and YY1 expression levels and survivorship in breast cancer patients, we studied a cohort that made available in the First Hospital of China Medical University. The characteristics of the studied cohort are summarized in Additional file 1: Table S1. After excluding cases with insufficient tumor tissue in tissue micro-array, *FEN1* expression was detectable in 268 cases, and *YY1* expression was detectable in 285 cases. The expression of FEN1 was detected in 209 cases out of a total of 268 cases (78.0%) by IHC staining, while YY1 was present in 67 cases out of a total of 285 cases (23.5%) by IHC staining. The association between *FEN1* and *YY1* expressions with clinicopathological variables of the cohort is shown in Additional file 1: Table S1. No significant association between FEN1 expression and age, T stage, N stage, stage, ER, PR, HR, Her-2, triple-negative, being ductal carcinoma in situ (Dcis), using taxane in adjuvant therapy, or using standard therapy was found. However, high YY1 staining correlated significantly with ER-positive cases (P = 0.007), PR cases (P = 0.000), HR-positive cases (P = 0.000), NOT-tri-negative cases (P = 0.030). The correlation between FEN1 and YY1 expression was not significant.

The 5-year overall survival rate of the cohort was 86.0%. In a Kaplan–Meier (KM) analysis, FEN1 and YY1 expressions showed no prognostic significance in OS in this cohort (P = 0.135 and 0.258, respectively). In contrast, patients with FEN1-high/YY1-low expression had significantly poor overall postoperative survival, compared with those with other phenotypes (P = 0.027) (Figure 6B). Stage was the only independent clinicopathological variable to predict OS (Additional file 1: Table S2.). After adjusting with the stage, FEN1-high/YY1-low expression could still predict a poor OS in the multivariate Cox model (P = 0.020, Additional file 1: Table S3). However, in the ER-negative or ER-negative/lymph-node-metastasis-negative subgroups, there was no significant association between the FEN1 expression level, YY1 expression level, or their combination and OS in the CMU cohort. The similar trends were observed in association between FEN1 expression, YY1 expression, their combination, and DFS when analyzed with KM methods. The corresponding log-rank P value was 0.102, 0.078, and 0.048, respectively. Patients with FEN1-high/YY1-low expression had significantly poor disease free postoperative survival (Figure 6C). Stage and Her2 status were independent clinicopathological variables to predict DFS (Additional file 1: Table S4). After adjusting with these 2 variables, FEN1-high/YY1-low expression could still predict a poor DFS in the multivariate Cox model (P = 0.007, Additional file 1: Table S5). However, in the ER-negative or ER-negative/lymph-node-metastasis-negative subgroup, there was no significant association among FEN1, YY1, or combination of the two and DFS.

## Discussion

Many chemotherapeutic drugs and ionized radiation have been employed to kill hyper-proliferating cancer cells, causing extensive DNA damage in the target cells. The DNA damage ultimately leads to cell cycle arrest and cell death. However, the efficacy of these therapeutic agents such as platinum drugs [53] and alkylating agents [54] can be significantly reduced by the ability of cells to repair their DNA. DNA repair involves an intricate network of repair systems that each targets a specific subset of lesions, including excision repair that replaces damaged or mismatched bases using the complementary strand as a template and homologous recombination and non-homologous end joining, both of which repair double strand breaks. An inverse correlation of ERCC1 (one of the nucleotide excision repair pathway components) with response either to platinum therapy or to survival was clearly established in ovarian cancer [55,56], non-small cell lung cancer [57] and colorectal cancers [58]. Moreover, mismatch repair (MMR) deficiency is associated with cisplatin resistance [59,60]. The MMR mechanism removes the newly inserted intact base instead of damaged base, triggering subsequent rounds of futile repairs, which can lead to cell death [61]. Furthermore, a role in triggering checkpoint signaling and apoptosis was also suggested [62]. Resistance to alkylating agents via direct DNA repair by O(6)-methylguanine methyltransferase (MGMT) has been extensively studied and is considered to be a significant barrier to the successful treatment of patients with malignant glioma [63]. In this study, we have presented data to support such a notion that FEN1 over-expression has an inverse correlation with survivorship of breast cancer patients and may serve as a prognostic biomarker. In molecular level, FEN1 expression is restricted by *FEN1* binding with transcription factor YY1. Upon treatment with cancer drugs, YY1 is released from the *FEN1* promoter so that the expression of FEN1 is highly induced in cancer cells, consequently leading to drug resistance. Therefore, the patients with low expression of YY1 and high expression of FEN1 have statistically significantly poor prognostic outcomes. It is worthy to note that DNA damage-induced FEN1 expression is at least partly mediated by the transcription factor p53 [29]. That FEN1 expression is repressed by YY1 is consistent with a previous study indicating that YY1 negatively regulate of p53 signaling [64].

Involved in DNA replication, DNA repair and apoptotic DNA fragmentation, FEN1 is a multi-functional nuclease, and the FEN1 level in human cells is tightly controlled. Previously, we have shown that FEN1 expression is controlled by DNA methylation at its promoter region [22]. In normal breast cells, the promoter region of *FEN1* is hypermethylated and the FEN1 level is low. However, during neoplastic transformation, this regulation mechanism is abolished, leading to FEN1 overexpression in breast cancer cells [22]. More recently, we have further demonstrated that the FEN1 protein level is also tightly controlled by sequential phosphorylation, SUMOylation, and ubiquitination in a cell cycle-dependent manner. Failure of these regulation processes may result in high FEN1 protein level, which is associated with abnormal cell cycle progression and genome instability [65]. Here, we reveal a new regulatory mechanism of FEN1. We demonstrate that *FEN1* gene expression is under control via binding of the transcription factor YY1 in the normal cell culture conditions. Relative large amount of the YY1 protein can be pulled down by the *FEN1* promoter DNA fragment containing the predicted YY1 binding motif that is conserved among various gene promoters. Three SNPs available in the database (NCBI SNP database http://www.ncbi.nlm.nih.gov/snp/?term=FEN1) beyond the conserved motif did not affect YY1 binding, while the nucleotide residue replacement in the conserved YY1 binding motif completely erased such a binding. We show that therapeutic drugs are able to disrupt the *YY1* gene expression and YY1 protein binding to FEN1 promoter region. It is known that YY1 is constitutively expressed in different types of cells, but its expression level also highly correlated with cell cycle progression and cell proliferation [66]. Therefore, it is possible that therapeutic drugs such as MMC and taxol induced cell cycle arrest and contribute to down-regulation of YY1. In addition, the drug treatment may induce changes in post-translational modifications and conformation of YY1 proteins or altering the FEN1 promoter methylation status, so that the interaction between *YY1* and *FEN1* promoter is impaired. As a result, the suppression of *FEN1* expression by YY1 is eliminated, and FEN1 is over-expressed. Consequently, cancer cells become more resistant to drugs due to enhanced DNA repair systems as a result of FEN1 over-expression. Moreover, after we artificially over-expressed YY1 protein, the cancer cells became more sensitive to drugs. Transcriptional factor binding sequence analysis of this region, using the computer programs TRANSFAC, Match1.0-public, TESS, and TFSEARCH, suggested that nearly 200 transcription factors including NF-κB and YY-1 might bind to the *FEN1* promoter region. Therefore, what we have seen with YY1 might be only tips of the iceberg. It is crucial to elucidate the comprehensive network that controls *FEN1* gene expression under both normal and treatment conditions and to obtain a dynamic picture on how such a control mechanism changes in response to different drug treatments.

From the clinical standpoint, FEN1 is a good candidate biomarker for prognostics of breast cancer patients based on evidences that we made available in the current studies. From the data made available by Ivshina [41], we see a clear distinction between the disease-free survivorship of the patients with high expression of FEN1 and that of patients with low expression of FEN1. Namely, more than 80% of patients with low expression of FEN1 can survive, disease-free, for more than 10 years; however, only less than 55% of patients with high expression can do so. That means 25% more patients in the cohort would live for at least 10 years longer if FEN1 expression were suppressed. The separation was very much further improved in ER- (45%) or ER- and lymph node negative patient cohorts (55%) though the cohort sizes are relatively small in the later two cases as only about 10% of breast cancer patients are ER-. In the patients with not only ER- but also low expression of FEN1 gene, more than 90% of them would be able to live for at least 10 years longer. Thus, examining the FEN1 expression level is very critical to the patients with ER-. With the patient cohort from the First Hospital of China Medical University, FEN1 and YY1 expressions were evaluated with IHC. In that cohort, patients with FEN1-high and YY1-low expression had both statistically significantly poor overall and disease-free postoperative survivals, a fact suggesting that FEN1 and YY1 might have inverse impact on the survival of breast cancer patients. This is consistent with the results that we have obtained from molecular studies using cultured cell lines and clinical drugs. Overall, the FEN1/YY1 interaction and regulatory mechanism might be of clinical importance and should be further investigated.

## Conclusion

Altogether, we demonstrate that YY1 plays a critical role in regulating FEN1 gene expression as a repressor. Reduction of YY1 levels in breast cancer cells results in overexpression of FEN1 leading to resistance to chemotherapeutic drugs. Conversely, overexpression of YY1 in the cancer cells suppresses FEN1 expression and sensitizes cancer cells to DNA damaging drugs. These finding provide basis for targeting YY1 and FEN1 for developing chemotherapy and radiation sensitizers.

## Additional file

Additional file 1: Table S1. Correlation between FEN1 or YY1 expression and clinicopathologic variables. **Table S2.** Univariate and multivariate analyses of OS according to clinicopathologic variables. **Table S3.** Univariate and multivariate analyses of OS according to biomarkers adjusted by selected clinicopathologic variables. **Table S4.** Univariate and multivariate analyses of DFS according to clinicopathologic variables. **Table S5.** Univariate and multivariate analyses of DFS according to biomarkers adjusted by selected clinicopathologic variables. **Table S6.** All the DNA oligonucleotides used in the current study.

## Abbreviations

BER: Base excision repair
ChIP: Chromatin immunoprecipitation
DFS: Disease-free survival
DNA: Deoxyribonucleic acid
EMSA: Electrophoretic mobility shift assay
ER: Estrogen receptor
FEN1: Flap endonuclease 1
HR: Homologous recombination
ICL: Interstrand crosslink repair
IHC: Immunohistochemistry
MDR: Multidrug resistance
MMC: Mitomycin C
MMR: Mismatch repair
NER: Nucleotide excision repair
NHEJ: Non-homologous end joining
NF-kB: Nuclear factor kappa-light-chain-enhancer of activated B cells
Nrf2: Erythroid-derived factor 2
PCR: polymerization chain reaction
SNP: Single nucleotide polymorphism
USF1: Upstream stimulatory factor 1
YY1: Yin Yang 1
