# Supplementary material for: YY1 suppresses FEN1 over-expression and drug resistance in breast cancer
Source: BMC Cancer. 2015 Feb 13;15:50. doi: 10.1186/s12885-015-1043-1 (PMC4348373; doi:10.1186/s12885-015-1043-1)
Supplement: Additional file 1: Table S1. — Correlation between FEN1 or YY1 expression and clinicopathologic variables. Table S2. Univariate and multivariate analyses of OS according to clinicopathologic variables. Table S3. Univariate and multivariate analyses of OS according to biomarkers adjusted by selected clinicopathologic variables. Table S4. Univariate and multivariate analyses of DFS according to clinicopathologic variables. Table S5. Univariate and multivariate analyses of DFS according to biomarkers adjusted by selected clinicopathologic variables. Table S6. All the DNA oligonucleotides used in the current study. [file 12885_2015_1043_MOESM1_ESM.pdf]

Supplemental Table S1. Correlation between FEN1 or YY1 expression and clinicopathologic variables

Variables	Total<br>n (%)	YY1 high, n (%)<br>high, n=285	P	FEN1 high, n (%)<br>high, n=268	P
Age years					
Median	51		0.384		0.251
Range	26-76				
T status					
T1	94(32.6)	22(23.4)	0.922	70(82.4)	0.305
T2-4	191(66.3)	45(23.9)		139(76.8)	
N status					
N0	130(45.1)	31(24.2)	0.747	96(80.0)	0.457
N+	156(54.2)	35(22.6)		112(76.2)	
Stage					
1&2	217(75.3)	54(25.2)	0.199	158(78.6)	0.82
3	68(23.6)	12(17.6)		51(77.3)	
ER					
Negative	161(55.9)	27(17.1)	0.007	120(77.9)	0.932
Positive	124(43.1)	38(30.6)		86(77.5)	
PR					
Negative	149(51.7)	18(12.2)	0.000	106(76.3)	0.616
Positive	133(46.2)	46(34.8)		97(78.9)	
HR					
Negative	121(42.0)	15(12.6)	0.000	88(76.5)	0.679
Positive	164(56.9)	50(30.7)		118(78.7)	
Her-2					
Negative	181(62.8)	48(27.0)	0.109	128(75.7)	0.24
Positive	104(36.1)	18(17.3)		80(81.6)	
Triple-negative					
Non- triple-negative	210(72.9)	56(26.8)	0.030	153(78.9)	0.575
Triple-negative	78(27.1)	11(14.5)		56(75.7)	
Taxane					
Never	205(71.2)	47(23.0)	0.768	148(77.5)	0.758
Ever	83(28.8)	20(24.7)		61(79.2)	
Standard therapy					
Standard therapy	253(87.8)	60(24.0)	0.791	187(79.6)	0.108
Non-std. therapy	32(11.1)	7(21.9)		20(66.7)	
Dcis					
Invasive	279(96.9)	66(23.9)	0.375	204(78.8)	0.099
Dcis	9(3.1)	1(11.1)		5(55.6)	
FEN1					
Low	59（20.5）	9（15.5）	0.089	-	-
High	209（72.6）	55（26.3）		-	
YY1					
Low	218（75.5)	-	-	154(75.9)	0.089
High	67(23.3)	-		55(85.9)	

Supplemental Table S2. Univariate and multivariate analyses of OS according to clinicopathologic variables

Biomarkers	Univariate			Multivariate		
	Hazard	95%Cl	P	Hazard	95%Cl	P
Age	0.993	0.963-1.024	0.674			
T stage	2.064	1.064-4.006	0.032			
N stage	3.184	1.675-6.051	0.000			
Stage	4.315	2.524-7.377	0.000	4.534	2.638-7.794	0.000
ER	0.699	0.401-1.218	0.206			
PR	0.650	0.373-1.130	0.127			
HR	0.708	0.411-1.220	0.214			
Her-2	1.716	1.063-2.768	0.027			
Triple-negative	1.135	0.623-2.068	0.678			
Taxane	1.287	0.721-2.298	0.393			
Standard therapy	1.561	0.758-3.217	0.227			
Dcis	0.047	0.000-36.425	0.368			

Supplemental Table S3. Univariate and multivariate analyses of OS according to biomarkers adjusted by selected clinicopathologic variables

Biomarkers	Univariate			Multivariate		
	Hazard	95%Cl	P	Hazard	95%Cl	P
FEN1-high	1.822	0.819-4.054	0.141	1.978	0.888-4.407	0.095
YY1-high	0.675	0.340-1.340	0.261	0.722	0.359-1.453	0.361
FEN1/YY1	1.944	1.065-3.546	0.030	2.06	1.121-3.782	0.020

Supplemental Table S4. Univariate and multivariate analyses of DFS according to clinicopathologic variables

Risk factors	Univariate			Multivariate		
	Hazard	95%Cl	P	Hazard	95%Cl	P
Age	0.989	0.965-1.013	0.370			
T stage	1.595	0.962-2.644	0.070			
N stage	2.570	1.574-4.197	0.000			
Stage	3.592	2.315-5.574	0.000	3.600	2.311-5.608	0.000
ER	0.892	0.573-1.386	0.610			
PR	0.802	0.515-1.250	0.330			
HR	0.880	0.568-1.365	0.569			
Her-2	1.687	1.149-2.476	0.008	1.515	1.003-2.288	0.048
Triple-negative	0.820	0.491-1.369	0.447			
Taxane	1.483	0.943-2.335	0.088			
Standard therapy	1.002	0.501-2.007	0.994			
Dcis	0.047	0.000-7.969	0.243			

Supplemental Table S5. Univariate and multivariate analyses of DFS according to biomarkers adjusted by selected clinicopathologic variables

Biomarkers	Univariate			Multivariate		
	Hazard	95%Cl	P	Hazard	95%Cl	P
FEN1-high	1.662	0.898-3.076	0.105	1.842	0.844-4.020	0.125
YY1-high	0.599	0.337-1.066	0.081	0.550	0.296-1.024	0.059
YY1/FEN1	1.988	1.221-3.235	0.006	2.085	2.421-5.997	0.007

Supplemental Table S6. All the DNA oligonucleotides used in the current study

Name	Sequence	Experiments
Probe a	5'-biotin-GGAGTTCATGGCAAGGGGCTTCCCC CTTCCCCACCCTTCAGCCCAAGCCGGAGGTTCCA -3	Biotinylated DNA pull-down assay
Probe b	5'-biotin-CGTCTGGAACTCCCCTCAACGCTCT CACCATTTTGCCCCGCGAAGGCTAATCCGCCGCT-3	Biotinylated DNA pull-down assay
Probe c	5'-biotin-GGCGCCTAGCACAACCGGAAAAGGAAG TGCCTCCG GCGCAAGTGGCATTGAGGGACTTG-3	Biotinylated DNA pull-down assay
Probe b_{SNP}	5'-biotin-CGTCTGGAACTCCCCTCAACGCTCT CTCCATCTTGTCCCGCGAAGGCTAATCCGCCGCT-3	Biotinylated DNA pull-down assay
Probe Random	5'-biotin-ATGCATGCATGCATGCATGCATGCA TGCATGCATGCATGCATGCATGCATGCATGCATG-3	Biotinylated DNA pull-down assay
YY1(pCDNA3.1(-)	Forward: GGTACC ATGGCCTCGGGCGACACCCT<br>Reverse: GATATC TCACTGGTTGTTTTTGGCC	Plasmid Construction
YY1(pCMV7.0-3*Flag)	Forward: GTCGAC GCCTCGGGCGACACCCTCTA<br>Reverse: TCTAGA TCACTGGTTGTTTTTGGCC	Plasmid Construction
pGL4.1-EGFP	Forward: AAGCTT GGCAATCCGGTACTGTTGGT AAAGCCACC ATGGTGAGCAAGGG<br>Reverse: TCTAGA TTACTTGTACAGCTCGTCCAT	Plasmid Construction
FEN1	Forward: TGGAGCGAGCCAAATGAA<br>Reverse: GCCGGTCACCTTGAAGAAA	qRT-PCR
YY1	Forward: CGACTACATTGAACAAACGCTG<br>Reverse: CGTTTCCCACAGCCTTCG	qRT-PCR
EGFP	Forward: CTTCTTGCCGTAGTTCCAC<br>Reverse: ACTGGGTGCTCAGGTAGTGG	qRT-PCR
GAPDH	Forward: GGGGAGCCAAAAGGGTCATCATCT<br>Reverse: GAGGGGCCATCCACAGTCTTCT	qRT-PCR
Probe N	5'-biotin-ATCGATCGATCGATCGATCGATCGATCG-3’	EMSA
Probe P(Probe b)	5'-biotin-CGTCTGGAACTCCCCTCAACGCTCTCACC ATTTTGCCCCGCGAAGGCTAATCCGCCGCT-3’	EMSA
WT FEN1	5'-biotin-AACGCTCTCACCATTTTGCCCCGCGAAG-3’	EMSA
MUT FEN1	5'-biotin-AACGCTCTCA*g*CA*a*TTTGCCCCGCGAAG-3’	EMSA
FEN1(YY1)	Forward: TGAGGAGATAAGCGCGGTGT<br>Reverse: AGGCACTTCCTTTTCCGGTT	ChIP/qRT-PCR
FEN1CDS	Forward: TGGAGCGAGCCAAATGAA<br>Reverse: GCCGGTCACCTTGAAGAAA	ChIP/qRT-PCR
ACTB	Forward: GCATAGCAGACATACAACGGAC<br>Reverse: GCACCTTTTACCCTGGAGC	ChIP/qRT-PCR
